# Coordinated transformation of the gut microbiome and lipidome of bowhead whales provides novel insights into digestion

**DOI:** 10.1038/s41396-019-0549-y

**Published:** 2019-12-02

**Authors:** Carolyn A. Miller, Henry C. Holm, Lara Horstmann, John C. George, Helen F. Fredricks, Benjamin A. S. Van Mooy, Amy Apprill

**Affiliations:** 1 Woods Hole Oceanographic Institution, Mailstop #4, 266 Woods Hole Road, Woods Hole, MA 02543 USA; 2 College of Fisheries and Ocean Sciences, University of Alaska Fairbanks, PO Box 757220, Fairbanks, AK 99775 USA; 3 Department of Wildlife Management, North Slope Borough, PO Box 69, Barrow, AK 99723 USA

**Keywords:** Microbiome, Animal physiology, Marine microbiology, Biogeochemistry, Microbial ecology

## Abstract

Whale digestion plays an integral role in many ocean ecosystems. By digesting enormous quantities of lipid-rich prey, whales support their energy intensive lifestyle, but also excrete nutrients important to ocean biogeochemical cycles. Nevertheless, whale digestion is poorly understood. Gastrointestinal microorganisms play a significant role in vertebrate digestion, but few studies have examined them in whales. To investigate digestion of lipids, and the potential contribution of microbes to lipid digestion in whales, we characterized lipid composition (lipidomes) and bacterial communities (microbiotas) in 126 digesta samples collected throughout the gastrointestinal tracts of 38 bowhead whales (*Balaena mysticetus*) harvested by Alaskan Eskimos. Lipidomes and microbiotas were strongly correlated throughout the gastrointestinal tract. Lipidomes and microbiotas were most variable in the small intestine and most similar in the large intestine, where microbiota richness was greatest. Our results suggest digestion of wax esters, the primary lipids in *B. mysticetus* prey representing more than 80% of total dietary lipids, occurred in the mid- to distal small intestine and was correlated with specific microorganisms. Because wax esters are difficult to digest by other marine vertebrates and constitute a large reservoir of carbon in the ocean, our results further elucidate the essential roles that whales and their gastrointestinal microbiotas play in the biogeochemical cycling of carbon and nutrients in high-latitude seas.

## Introduction

As the largest animals in the ocean, each feeding on tons of smaller prey each day, whales are a stabilizing force in the global ocean ecosystem. Their digestive processes not only support their residence in dynamic and often extreme ocean conditions, but also contribute to the ocean’s biogeochemical cycles [1–3]. In harvesting nutrients and energy from the enormous quantities of prey they consume, whales transform, concentrate, and release scarce nutrients into the water column, which, in turn, stimulate primary production. Indeed, whales and seals replenish more nitrogen into the photic zone of the Gulf of Maine per year than the input of all rivers combined, ~2.3 × 10^4^ metric tons [2]. Also, the defecation of the trace micronutrient iron by sperm whales in the Southern Ocean stimulates primary production that drives the export of 2 × 10^5^ metric tons carbon to that ocean per year [1]. Thus, regional-scale impacts on the cycling of iron and nitrogen by whales can lead to basin-scale impacts on the carbon cycle. In this way, whale digestion can be considered fundamental to the hierarchy of processes that move energy, nutrients, and organic matter throughout the ocean’s food web. Despite the importance of whale digestion to the oceans, it is poorly understood.

Adequate nutrition is essential for many aspects of mammalian physiology, including thermoregulation, immune function, and reproduction, and ultimately, survival of a species. This is particularly true for baleen whales, for which ample body fat reserves are critical for sustaining them during periods of fasting (e.g., during migration and, for females, the initial months of lactation) and maintaining body temperature in colder waters, among other reasons. Baleen whales consume prey rich in high-energy lipids, such as wax esters and triglycerides. These molecules provide energy to replenish or maintain body fat reserves, as well as to sustain general metabolism. At times, wax esters can comprise up to 94% of the lipids consumed by whales [4, 5]. While digestion of wax esters is considerably slower than that of triglycerides in fish [e.g., 6, 7] and somewhat less efficient in terrestrial mammals [e.g., 8], wax ester digestion appears to be highly efficient in at least two species of large whales [4, 9]. However, the mechanism(s) for digestion of wax esters and other lipids by whales is currently unknown. Given the major role wax esters have in the ‘energy economy’ of many marine animals and that estimations indicate that at times, wax esters store at least half of the carbon produced by primary production in the oceans world-wide [10], it is of great interest to elucidate how whales are contributing to the cycling of this important marine lipid.

Mammalian digestion, through the actions of the gastrointestinal (GI) tract, involves breaking down, extracting, and absorbing energy and nutrients from food, and removing waste products. The anatomy of the whale GI tract is well described. As an alimentary canal designed to digest prey that is swallowed intact [11], the whale GI tract comprises four stomach chambers, an initial nonglandular compartment connected to three glandular chambers, followed by a mucous lined sac that opens into a typical mammalian small and large intestine [12, 13]. In contrast, less is known about the gut microbiotas of whales. Early studies of harvested baleen whales suggested microbial fermentation occurs in the forestomach, and that the forestomach and colon host anaerobic bacteria [14, 15]. More recent culture-independent methods described microorganisms from baleen whale fecal samples, identifying connections to diet (carnivore, herbivore) and host phylogeny [16]. The gut microbiota often has metabolic capabilities that are not encoded in the host genome, including the capacity to degrade otherwise indigestible components of the diet [17]. In this regard, the gut microbiota may contribute to digestion of wax esters and other lipids in baleen whales. However, the general inaccessibility of whales has limited the ability to characterize the gut microbiota of whales and its connection to lipid digestion.

To ascertain the possible connection of the whale gut microbiota to the digestion of essential constituents of the whale diet, i.e., lipids, we examined the gut microbiota and lipidome in digesta collected from nine distinct anatomical regions of the GI tract, from forestomach to large intestine, of bowhead whales (*Balaena mysticetus*) harvested during Native Alaskan subsistence hunts. Bowhead whales are baleen whales belonging to the family Balaenidae. They have the thickest blubber layer of all species of whales, can reach lengths of up to 18 m, and reside entirely in Arctic and sub-Arctic oceans [12, 18]. Our results revealed that the microbiota and lipidome were highly correlated throughout the GI tract. The richness of the microbiotas was lowest in the stomach chambers, where core bacterial groups were anaerobic, and highest in the large intestine, where the microbiotas were most similar among whales. The abundance of wax esters, the primary lipids in *B. mysticetus* prey representing more than 80% of prey lipids, was significantly decreased in the distal small intestine (by more than 50%), and strongly suggests that this important marine lipid, which is difficult to digest by most mammals and fish, is digested in the mid- to distal small intestine. Our results also suggest that the small intestine microbiotas, including specific bacterial taxa, might be involved in the digestion of wax esters. Thus, by characterizing the biogeography of the microorganisms and lipids throughout the GI tract, our results offer a unique view of digestion in a species of baleen whale whose Arctic-based lifestyle is dependent on efficient lipid digestion.

## Materials and methods

### Sample collection

**Fig. 1:**
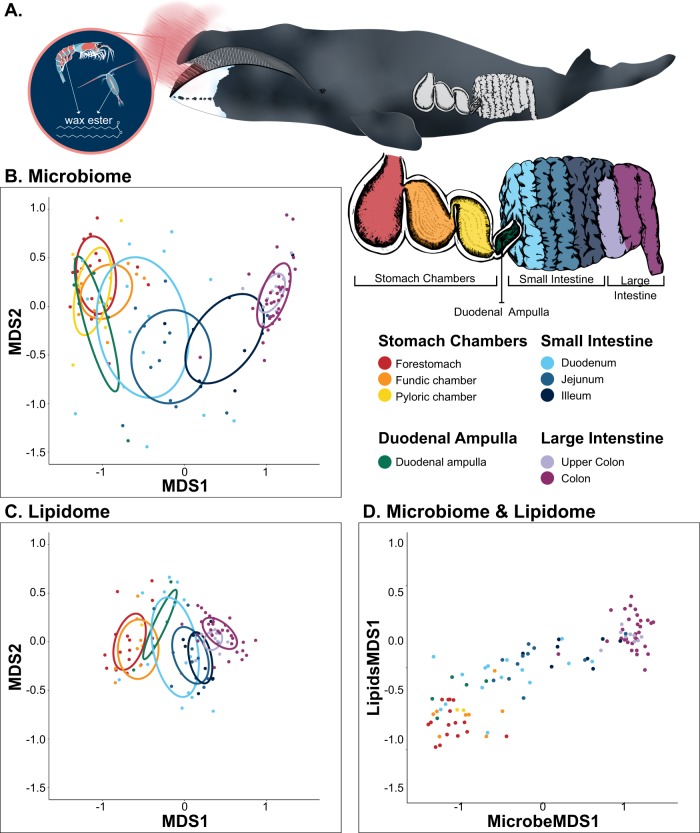
Comparison of the bowhead whale microbiome and lipidome across the gastrointestinal (GI) tract of bowhead whales. **a** Illustration of a bowhead whale feeding on zooplankton (inset: copepod and krill) and the nine GI areas from which samples of GI contents were collected. Nonmetric multidimensional scaling (nMDS) comparisons of Bray–Curtis dissimilarities of **b** microbial communities based on the 361 minimum entropy decomposition nodes [23] (MED nodes) (*n* = 121) and **c** the 546 lipids (*n* = 106). For both figures, dispersion ellipse centroids are defined by the mean dissimilarities for each anatomical location and the ellipse shapes are defined by the covariances. **d** Comparison of MDS1 scaling from the paired microbiome and lipidome samples across the GI tract (*n* = 105)

Digesta (luminal contents) were opportunistically collected from up to nine anatomical locations in the GI tracts of 38 bowhead whales, 20 females and 18 males, harvested during the fall Native Alaskan subsistence hunts in Utqiaġvik, AK, USA, 2009 and 2011–2013 (Table S1). The nine GI sampling locations comprised three stomach chambers, four locations in the small intestine and two locations in the large intestine (Fig. 1A). Stomach chambers sampled were (*n* microbiota, lipidome) the forestomach (18, 16), the fundic (10, 8), and the pyloric (8, 2) chambers. Small intestine samples were collected from the duodenal ampulla (the chamber that connects the pyloric stomach chamber with the small intestine) (5, 5), duodenum (16, 15), jejunum (14, 13), and ileum (9, 8). Large intestine samples were collected from the proximal colon (10, 10) and mid-lower colon (36, 33). Not all nine locations were sampled from each whale. Some GI tract locations had insufficient material for sampling because bowhead whales do not feed continuously and digesta from discrete feeding events separately pass through the GI tract. Samples were collected ~8–15 h postmortem, which is significantly less time than samples collected during necropsies of most large whales. Seawater and air temperatures during the towing of the whale to the butchering site and sample collection were near freezing, which helped preserve the quality of the samples. For microbiota analysis, digesta was collected in sterile 2 mL cryovials, frozen in a liquid nitrogen vapor shipper, and transferred to −80 °C until processing. For lipidome analysis, digesta was collected in 50 mL centrifuge tubes and frozen at −20 °C until processing.

### Nucleic acid preparation and sequencing

Nucleic acids were isolated from 50 mg of each sample using the PowerFecal DNA Isolation Kit (MO BIO Laboratories, Inc., Carlsbad, CA, USA). The V4 region of the SSU rRNA gene was amplified in triplicate 25 μL PCR reactions using the 515F and 806RB primers [19, 20] as described by Apprill et al. [21] except that differential PCR cycles (18–38) were used to optimize amplification to account for any potential unevenness of microbial amplification among samples from different GI tract locations. To test for amplification bias and sequencing errors, two low-concentration microbial mock communities, one with equimolar and one with staggered ribosomal RNA operon counts (BEI Resources, NIAID, NIH, Manassas, VA, USA, as part of the Human Microbiome Project: Genomic DNA from Microbial Mock Community B, HM-782D and HM-783D, v5.2 L) were also amplified. For each sample, the triplicate products were combined, purified using the Agencourt AMPure XP system (Beckman Coulter, Inc, Danvers, MA, USA), and quantified using the Qubit 2.0 Fluorometer with a dsDNA High Sensitivity Assay kit (Invitrogen, Carlsbad, CA, USA). Purified amplicons were pooled in equal concentrations and sequenced using the paired end, 2 × 250 bp MiSeq Illumina format at the University of Illinois W. M. Keck Center for Comparative and Functional Genomics using the approach described by Kozich et al. [20]. Each PCR run included sterile water as a negative control, for which amplification was not detected for any of the runs. A representative negative control was sequenced but the minimal number of reads did not pass quality control (QC) during the denoising and quality filtering of sequences described below.

### Sequence analysis

Mothur v.1.33.3 [20] was used to assemble, denoise, and quality filter the raw reads with UCHIME [22] for chimera detection. Reads identified as chimeras (0.42% of the dataset) were removed. The dataset was subsampled to 12,000 reads per sample to minimize any effect from read count variation. Minimum entropy decomposition (MED) [23] was used to assign 1,728,000 sequences to operational taxonomic units, herein referred to as MED nodes. For the MED, minimum substantive abundance was set to 172 (the number of reads in the dataset divided by 10,000) according to the recommendations of Eren et al. [23]. Representative sequences from each MED node were classified using a k-nearest neighbor consensus algorithm in Mothur with the Silva ribosomal RNA sequence database (v.132) [24]. Similarity to other sequences was assessed using nucleotide similarity percentages from standard nucleotide BLASTN 2.8.1 metablast optimized searches [25, 26] applied to the National Center for Biotechnology Information nucleotide database collection or with specific BLASTN sequence alignments. Sequencing error rate calculated from the mock communities was 0.002%. See Supplementary Methods for further details.

### Lipid extractions

Total lipid extracts (TLEs) were prepared using a modified Bligh and Dyer method [27, 28]. For each sample, 0.8 g of wet content was weighed into a 7 mL glass vial and 10 μL of a 1.5 mmol L^−1^ solution of butylated hydroxy toluene was added as an antioxidant. Samples were homogenized three times using a tissue homogenizer (Omni International, Kennesaw GA): prior to, between, and after the addition of the initial 2 mL methanol and 1 mL dichloromethane (DCM) extraction solvents to the vial. The TLEs were stored under argon in 2 mL high performance liquid chromatography (HPLC) vials at −20 °C until analysis. A pooled sample was prepared from aliquots of all TLEs for use as a QC. The TLEs and the pooled QC were diluted 1:50 with DCM. All glassware was combusted prior to use.

### Liquid chromatography–mass spectrometry analysis

Extracted lipids were analyzed by HPLC–electrospray ionization—mass spectrometry (HPLC-ESI-MS) on an Agilent 1200 HPLC (Agilent Technologies, Santa Clara, CA, USA) coupled to a Q Exactive Hybrid Quadrupole–Orbitrap mass spectrometer (Thermo Fisher Scientific, Waltham, MA, USA) as described by Collins et al. [29] (see also Supplementary Information). A Nova-Pak C18, 4 μm, 3.9 × 150 mm HPLC column (# WAT086344, Waters Corp. Milford, MA, USA) was used with gradient elution as shown in Tables S2 and S3. Samples were kept at 5 °C in the autosampler prior to analysis. Injection volume onto the column was 2 μL. The pooled QC aliquot was run after every eight samples.

### Lipid identification and downstream analysis

Lipid compound identification in mass spectrum data was assisted by a pipeline the of open-source R programming packages xcms [30–32], CAMERA [33], and LOBSTAHS [29]. This pipeline allows for high-throughput annotation and putative identification of mass features selected using an array of criteria. Mass features are detected, grouped, and retention time corrected by xcms and CAMERA, with final lipid species annotations proposed by LOBSTAHS. Final identifications were manually confirmed using MS^2^ spectra, retention time patterns, and accurate mass. Relative abundances of lipids were corrected for ionization response after calculating the response factors of representative external standards (Table S4). The size of triacylglycerols (TAG) is known to cause inconsistencies in ionization response; thus, TAG peaks were corrected according to Holčapek et al. [34] with a range of different sized TAGs standards (Nu-Check-Prep, Inc., Elysian, MN, USA).

### Data analysis

Lipidome and microbiota data were analyzed using RStudio [35] for R [36], Primer 7 software (PRIMER-E, Ltd, Plymouth, UK) and the PERMANOVA add-on to Primer 7 (PRIMER-E, Ltd, Plymouth, UK) (see Supplementary Information for details). Only the samples for which both lipidomes and microbiotas were analyzed were included in the direct comparisons of the lipidomes and microbiotas. Nursing calves were excluded from all statistical analyses.

## Results

Sequencing of the V4 region of the SSU rRNA gene on the digesta (luminal contents) samples produced only sequences affiliated with the domain Bacteria. MED [23] grouped the sequences into 361 MED nodes based on Shannon entropy decomposition of information-rich nucleotide positions, which is similar to a fine-scale microbial taxonomic designation. Nonmetric multidimensional scaling (nMDS) of Bray–Curtis dissimilarity indices of the MED nodes demonstrated significant partitioning of the bacterial communities according to anatomy (Fig. 1B) (PERMANOVA: Pseudo-F = 12.803, SS = 1.693E + 05, df = 8, *P* = 0.001). Pairwise comparisons of the microbiotas between anatomical locations showed that communities were most different among three main regions: stomach, small intestine, and large intestine, with sub-regional differences also detected in the small intestine (Table S5).

The lipidome comprised 546 lipids. Like the microbiota, nMDS of Bray–Curtis dissimilarity indices of the lipidome demonstrated significant partitioning with GI tract anatomy (Fig. 1C; PERMANOVA: Pseudo-F = 10.452, SS = 49000, df = 7, *P* = 0.001) with pairwise comparisons also showing that the three main regions of the GI tract, stomach, small intestine, and large intestine, were most different, with sub-regional differences in the small intestine (Table S6).

Comparison of the first factors of the nMDSs of the microbiota and lipidome revealed a strong correlation between the datasets (Spearman’s rank correlation: *r*_*s*_ = 0.85, *S* = 28722, *P* < 0.0001) that also varied with the anatomy of the GI tract (Fig. 1D). A Mantel test for correlation between the two matrices confirmed that the community compositions of the microbiotas and lipidomes were significantly correlated (Mantel test: *r*_*s*_ = 0.5142, *P* = 0.001).

### Microbial community composition changes and diversifies in the intestine

**Fig. 2:**
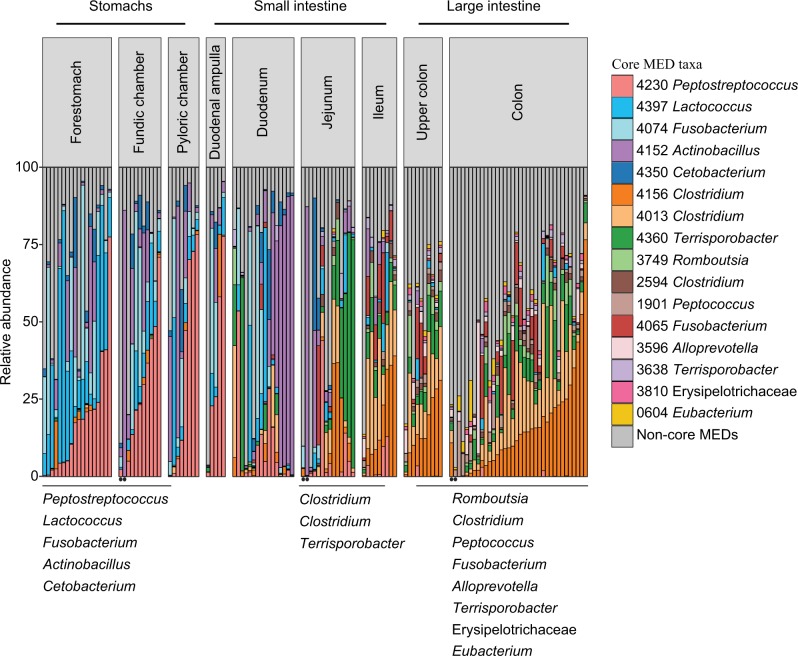
Relative abundance of minimum entropy decomposition (MED) nodes identified as core members (taxa present at greater than 1% abundance in more than 50% of samples) within each gastrointestinal (GI) tract sampling location for 121 samples of GI contents collected from 38 bowhead whales. Areas of the GI tract in which the taxa emerged as core are shown beneath the *x*-axis. Circles denote samples from nursing calves

Bacteria associated with nine phyla were observed in all GI tract locations but 97% of the sequences were assigned to four phyla: Firmicutes (64%), Proteobacteria (16%), Fusobacteria (12%), Bacteroidetes (5%). While Firmicutes were dominant throughout the GI tract, Proteobacteria, and Fusobacteria were most abundant in the proximal GI tract, and Bacteroidetes emerged in the large intestine (Fig. S1). On a finer scale, sharp shifts occurred in bacterial composition throughout the GI tract locations, which was primarily driven by 16 distinct MEDs (MED nodes with relative abundances >1% in >50% of the samples in each GI location; herein referred to as ‘core’ groups) (Fig. 2). Five of the 16 core bacterial groups were common to the three stomach chambers: *Fusobacterium* (MED4074), *Cetobacterium* (MED4350), *Peptostreptococcus* (MED4230), *Lactococcus* (MED4397), and *Actinobacillus* (MED4152). All but *Fusobacterium* (MED4074) persisted into the duodenum. The jejunum appeared to be an area of transition for these 16 core groups. While three of the core groups found in the proximal half of the GI tract persisted into the jejunum (MEDs 4230, 4152, 4397), three additional Clostridiales-affiliated groups common to the distal half of the GI tract also were present in the jejunum, *Clostridum* (MEDs 4156 and 4013) and *Terrisporobacter* (MED4360). The number of these core bacterial groups detected in the distal half of the GI tract increased as the gut progressed, with six in the jejunum, seven in the ileum, and 11 in the large intestine. The large intestine-associated core groups were primarily affiliated with the order Clostridiales: *Clostridium* (MEDs 4156, 4013, and 2594), *Romboutsia* (MED3749), *Terrisporobacter* (MEDs 4360 and 3638), *Peptococcus* (MED1901), and a group belonging to Family XIII (MED604). Other large intestine core groups were *Fusobacterium* (MED4065), *Alloprevotella* (MED3596), and Erysipelotrichaceae (MED3810).

**Fig. 3:**
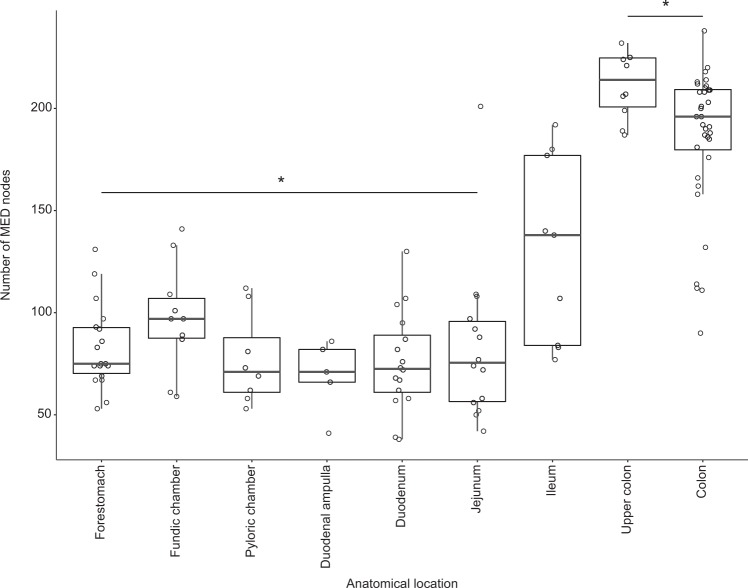
Microbial community richness, the number of observed minimum entropy decomposition (MED) nodes, in 121 samples of gastrointestinal (GI) contents collected from 38 bowhead whales. Five summary statistics are visualized in the boxplot: median, the two hinges, which correspond to the 25th and 75th percentiles (the first and third quartiles), and the upper and lower whiskers, which extend from the upper and lower hinges to the largest and smallest values no further than 1.5 times the inter-quartile range (the range between the hinges). Asterisks indicate the forestomach through the duodenum are significantly different from both large intestine sites (*P* < 0.02)

More broadly, the total number of microbial taxa (MED nodes) was significantly greater (a two-fold difference) in the large intestine (192 ± 33) when compared with the proximal and mid-GI tract (stomachs, duodenal ampulla, duodenum, and jejunum: 82 ± 27) (Kruskal–Wallis rank sum test *Χ* = 86.036, df = 8, *P* < 0.0001; Dunn post-hoc tests *P* < 0.02; Fig. 3). Moreover, the bacterial communities of the large intestine were more similar among whales (58%) than those in other GI tract locations (28–48%; Fig. 1B and Table S7).

Bray–Curtis dissimilarity indices of the MED nodes demonstrated significant partitioning of the bacterial communities according to sampling year nested within GI tract sampling location (PERMANOVA: Pseudo-F = 1.3477, SS = 44680, df = 22, *P* = 0.019; Table S5). Pairwise comparisons among years nested within anatomical locations showed that bacterial communities from whales sampled in 2009 were significantly different from those sampled in 2011 and 2013 (*P* *<* 0.03) and whales sampled in 2011 differed from those sampled in 2013 (*P* = 0.015; Table S5).

### Evidence suggesting digestion of main prey lipids occurs in the small intestine

**Fig. 4:**
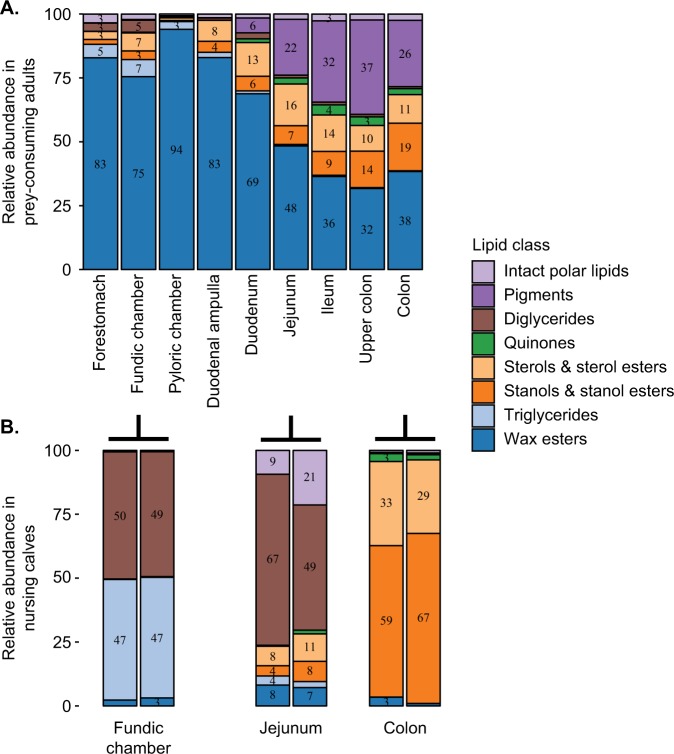
Abundances of lipid classes relative to total lipids within each gastrointestinal (GI) tract sampling location in bowhead whales, pooled for 112 samples of GI contents collected from **a** 35 prey-consuming juvenile and adults and **b** two nursing calves. Numbers in the bars denote the percent pooled relative abundances of the lipid classes

The 546 lipids detected throughout the GI tract of the whales belonged to eight classes: wax esters, triglycerides, sterols and sterol esters, stanol and stanol esters, quinones, diglycerides, pigments, and intact polar lipids. Wax esters and triglycerides, abundant in the prey of bowhead whales, dominated the proximal half of the GI tract, while sterols and sterol esters, stanols and stanol esters, quinones, and pigments (primarily astaxanthin) were abundant in the distal half of the GI tract (Fig. 4A). The small intestine, particularly the jejunum, was the area that delineated these major differences in lipid composition (Fig. 4A, Fig. S2).

**Fig. 5:**
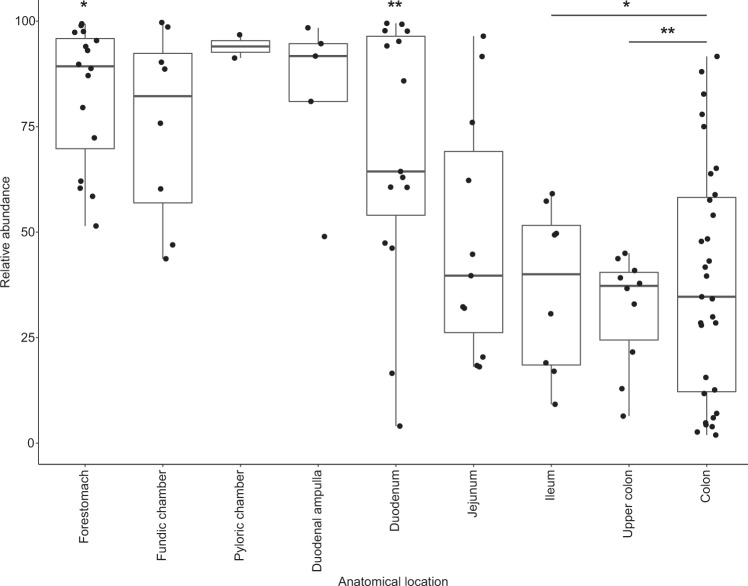
Abundances of the wax ester lipid class relative to total lipids in 106 samples of gastrointestinal contents collected from 35 bowhead whales. Five summary statistics are visualized in the boxplot: median, the two hinges, which correspond to the 25th and 75th percentiles (the first and third quartiles), and the upper and lower whiskers, which extend from the upper and lower hinges to the largest and smallest values no further than 1.5 times the inter-quartile range (the range between the hinges). The single asterisk indicates the forestomach is significantly different from the ileum, upper colon, and colon (*P* < 0.001). Double asterisks indicate the duodenum is significantly different from the upper colon and colon (*P* < 0.03)

Wax esters were the most abundant lipids in the bowhead GI tract, particularly in the stomach chambers, and appear to be digested in the mid- to distal small intestine. Mean relative abundance of the wax ester lipid class was greatest in the stomachs and duodenal ampulla (the chamber at the start of the small intestine) and steadily decreased throughout the small intestine, reaching the lowest abundance in the ileum and proximal large intestine (Figs. 4A and 5). The distributions of the relative abundance of the wax esters differed significantly among anatomical locations (Kruskal–Wallis rank sum test *Χ* = 42.85, df = 7, *P* < 0.0001; Fig. 5) with pairwise comparisons revealing a significant decrease (by more than 50%) between the first stomach chamber (mean ± 1 SD, *n*: 82.85 ± 16.49%, 16) and the distal small intestine (ileum: 36.44 ± 19.81, 9) and the large intestine (proximal colon: 31.73 ± 13.42%, 11; mid-lower colon: 38.39 ± 27.81, 34) (Bonferroni adjusted Dunn post-hoc tests *P* ≤ 0.03; Fig. 5).

Total wax ester abundance decreased significantly between the forestomach and colon in the 14 whales sampled in both locations (Paired *T*-Test: *t* = 5.7426, df = 13, *P* = 3.396e−05; Fig. S3A). Although the paired test showed a significant decrease in total relative abundance of wax esters between forestomach and colon, the difference in abundance of the wax esters between these two locations was highly variable among the 14 whales (mean ± 1 SD, range: 46.5 ± 30.3%, 4.4–91.3%; Fig. S3B). Moreover, the relative abundance of wax esters still present in the colon also was highly variable, with a mean ± 1 SD, 38.39 ± 27.81% and range from 1.94 to 91.63% (Fig. 5).

In comparison with wax esters, triglycerides were far less abundant in the stomach chambers. Like wax esters, distributions of the total abundance of triglycerides relative to total lipid abundance differed significantly among anatomical locations (Kruskal–Wallis rank sum test *Χ* = 45.36, df = 7, *P* < 0.0001; Figs. S2 and S4) with pairwise comparisons revealing significant differences between the proximal GI tract (stomach chambers and duodenal ampulla) and the large intestine (Bonferroni adjusted Dunn post-hoc tests *P* ≤ 0.05). Mean relative abundance of triglycerides was greatest in the stomach chambers and decreased by half for each GI location after the stomachs to the lowest observed amount in the colon.

With regards to the other major lipid classes (Fig. S2), the relative abundances of the sterols and sterol esters increased in the small intestine and decreased slightly in the large intestine, whereas stanols and stanol esters gradually increased from the duodenal ampulla to the colon. Quinones, of which ubiquinone was the most abundant, first appeared in the duodenum and were most abundant in the ileum and large intestine. Likewise, the pigment astaxanthin was abundant in the distal half of the GI tract. The relative abundance of intact polar lipids was consistently low throughout the GI tract, although, phosphatidylcholine was present only in the proximal half of the GI tract. The relative abundance of diglycerides, although relatively low compared with the total lipid content, was most abundant in the stomach chambers for the prey-consuming adult and juvenile whales (Fig. 4A).

### Nursing calves exhibit distinct lipidomes and microbiotas

Interestingly, two bowhead whale calves had milk in their stomachs and their intestinal content lipid profiles were very different from all other prey-consuming whales in the study (Fig. 4B). The lipids in the stomachs of these two whales were dominated by triglycerides (47% each) and diglycerides (49 and 50%), with small amounts (2 and 3%) of wax esters also present. While the presence of milk in the stomachs suggests these two whales were nursing calves, the presence of small amounts of wax esters might suggest that they were beginning to feed on prey as well. The lipids in the mid-small intestine (jejunum) were dominated by diglycerides (40 and 57% of the total lipids), while those in the colon were dominated by sterols, sterol esters, stanols, and stanol esters. The microbial profiles of the calves also were different from those of all other whales. The nMDS of Bray–Curtis dissimilarity indices of the microbiotas of all whales showed that the indices of the calves were outliers; they were located outside of the covariance ellipses for the three GI tract locations sampled (Fig. S5). Also, the abundances of the 16 core members of the adult microbiotas appeared different in the calves, with *Actinobacillus* (MED4152) dominating the microbiotas of the fundic chamber and jejunum in one of the calves (Fig. 2).

### Microbes correlate with wax ester abundance in the mid-small intestine

The distance matrices of the microbiotas and the wax ester pools were correlated in the mid-small intestine (jejunum), the location where wax esters decreased (Mantel test: *r*_*s*_ = 0.5109, *P* = 0.003). Also, two of the core MEDs, *Actinobacillus* (MED4152) and *Cetobacterium* (MED4350), correlated with total relative abundance of wax esters in the jejunum (Spearman’s rank correlation: *r*_*s*_ = 0.78 (MED4152), 0.72 (MED4350), FDR adjusted *P* = 0.04, *n* = 11; Fig. S6).

## Discussion

To investigate the digestive capabilities of a baleen whale species, we present an in-depth examination of the links between gut microbes and lipid digestion throughout the GI tract of bowhead whales. Using high-resolution surveys of lipid and microbial communities, we demonstrate that the composition of the lipid and bacterial communities change in a highly correlated manner in the nine locations across the GI tract. The most significant changes in lipids and specific bacterial groups occurred within the small intestine, followed by a diversification of the microbiota in the large intestine. We provide evidence suggesting digestion of an important marine lipid, wax esters, in bowhead whales and that certain microorganisms may contribute to that digestion. We further show that lipids and GI bacteria are distinct in nursing calves. These GI biogeographic-based results of the bowhead whale lipidome and microbiota contribute to our understanding of the significance of lipid digestion and whale GI tract-associated microbes to the ocean ecosystem.

### Lipids and microbes exhibit similarity across the stomach chambers

Bowhead whales, like other whales, have a multi-chambered stomach that somewhat resembles that of cows, pigs, and other terrestrial foregut fermenters, to which whales are phylogenetically related [37]. After passage through an esophagus, prey that was filtered from the seawater by baleen and swallowed intact enters the first stomach chamber, the forestomach (mechanical digestion), followed by the fundic and pyloric chambers (mechanical and chemical digestion) [12]. Despite the different physical and chemical environments of the chambers, both the lipidomes and the microbiotas were highly similar among stomach chambers of individual whales. This high degree of similarity may, in part, reflect mixing/reflux of digesta through the relatively large orifice connecting the forestomach and fundic chamber [12].

Wax esters were the most abundant lipid in the bowhead stomach chambers, accounting for 51–99% of the total lipids. These wax esters ranged in size from 30 to 44 carbon atoms with 1–7 double bonds. Triglycerides, in contrast, only accounted for 0.1–20% of the total lipids. Bowhead whales primarily feed on calanoid copepods (mainly *Calanus hyperboreus* and *C. glacialis*) and euphausiids (krill; mainly *Thysanoessa raschii* and *T. inermis*), but other crustaceans, such as mysids, amphipods, and isopods, and fish, were also found in the stomachs of harvested whales [38, 39]. In *Calanus* spp., long-chain (38–44 carbon atoms) di- and polyunsaturated wax esters accounted for 68 to more than 90% of the total lipids whereas triglycerides only accounted for 1–8% [40–42]. In krill, shorter-chain (30–34 carbon atoms) unsaturated and monounsaturated wax esters accounted for 10 to 40% of the total lipids, while triglycerides accounted for 28 to 44% [42]. Thus, the lipidomes we observed in the stomachs more closely reflected the copepod prey.

The microbial communities in the bowhead stomach were characterized by low community richness, as would be expected in an acidic stomach environment. The bacteria we identified in the stomach were similar to those previously isolated from the GI tracts of other cetaceans [43–45] (see Supplementary Discussion for details). Overall, the core bacterial groups we identified in the bowhead whale stomach chambers were anaerobic taxa, and their distribution among diverse cetaceans may suggest a common role in digestion in the cetacean stomach.

### Wax ester digestion and major changes to microbes in the small intestine

In mammals, the majority of chemical digestion and absorption of food occurs in the small intestine. The mammalian small intestine comprises the duodenum (proximal), the site of chemical digestion, jejunum (mid), the site of nutrient absorption, and ileum (distal), the site of absorption of bile salts, vitamin B12, and other products of digestion that were not absorbed in the jejunum. The small intestine of the bowhead whale is typical of a mammal with a duodenum, jejunum, and ileum, but it also has a dilated sac at the beginning called the duodenal ampulla, which is thought to be a mixing chamber [12].

Bacterial community composition was highly variable in each of the three areas of the small intestine; yet, the number of bacterial taxa comprising those communities remained low in the proximal and mid-small intestine when compared with the large intestine. Little is known about the physicochemical properties that could affect the microbial communities in the small intestine of whales except that in minke whales, the pH was low in the proximal regions and increased to neutral in the ileum [11]. It may be that the conditions in the small intestine of bowhead whales are similar to those in other mammals that result in less stable, less diverse, and lower density microbial communities [46–51]. With the relatively long length of the small intestine and general lack of distinctive features marking the transitions between the three areas, it is possible that there were inconsistencies in the sampling locations, which also may have contributed to the high variability in community composition among individual whales.

Wax esters decreased significantly between the stomach and distal small intestine, suggesting wax ester digestion occurs in the mid- to distal small intestine of bowhead whales. The ability to digest wax esters appears to vary across vertebrate species: two species of baleen whales [4, 9] and certain seabirds were reported to be highly efficient, ~90% and 85%, respectively, whereas rats and dogs were less efficient, 50% and 10%, respectively [8, 52]. Wax esters hydrolyze at a slow rate, up to 25 times slower than triglycerides in multiple species of marine fish [6, 7, 53–56], due, in part, to their high degree of hydrophobicity. Studies on wax ester digestion in fish and seabirds suggested increased retention time of wax esters in the GI tract is one factor that may be necessary for increased hydrolysis of wax esters [55, 57]. Our results showed that unlike triglycerides, which decreased substantially in the proximal intestine, wax esters did not reach their lowest abundance until the end of the small intestine. Hence, wax esters, either because of the large quantity consumed or because of their chemical properties, may need more time and/or exposure to the physicochemical/biological properties of the small intestine for digestion in bowhead whales.

The mechanism for wax ester digestion in whales is unknown. In fish, wax ester hydrolysis was attributed to the enzyme called wax ester hydrolase, which appears to be dependent on bile salts [7, 58]. In seabirds, the high assimilation rates of wax esters were associated with high concentrations of bile salts, reflux of digesta (gastric and duodenal) into the gizzard for furthers emulsification, and almost equal rates of triglyceride and wax ester hydrolysis [52]. Although it has been proposed that microbes may aide in the digestion of wax esters, the evidence from previous studies has been contradictory [59, 60]. Here, in the jejunum of bowhead whales, the location where wax ester abundance was decreasing, we observed a correlation between wax esters and the microbiotas. Also, two of the core bacterial taxa, *Actinobacillus* (MED4152) and *Cetobacterium* (MED4350) were correlated with wax ester abundance in the jejunum. As enzymes for wax ester hydrolysis are currently uncharacterized in both bacteria and whales, understanding the specific contribution of the bowhead whales and their microbes to wax ester digestion will necessitate greater insight than revealed by sequencing-based methods, such as direct experiments with bacterial isolates.

### Diversification of microbes coincides with reduced prey lipids in the large intestine

The large intestine (proximal colon and colon) was the site of highest microbial community similarity among bowhead whales, compared with the other locations in the GI tract. These highly conserved microbial communities also were significantly more species rich (i.e., greater number of MEDs) than all other GI locations except the ileum, in which moderately enhanced microbial richness was observed. Similarly, the bacterial communities in the large intestine of humans were more diverse than those of the small intestine [47, 48]. Physicochemical factors influencing microbial communities in the large intestine of humans and other mammals include a slower luminal flow (resulting in a longer retention time), lower concentrations of bile salts, and less acidic pH than in other GI locations, and the lymph tissue that monitors intestinal bacteria in the small intestine is absent [46, 49]. A diversification of the core members of the large intestine microbiota was also observed, with 11 of the 16 total GI core bacterial groups (MEDs) present in the upper (proximal) colon. Many of the same bacterial taxa that were core members of the mid- and distal small intestine microbiotas were also core members of the large intestine (*Clostridium*, *Terrisporobacter*, *Romboutsia*, *Fusobacterium*), but new taxa associated with *Eubacterium*, *Peptococcus*, Erysipelotrichaceae, *Alloprevotella*, and an additional *Terrisporobacter* also emerged as core members. Despite the high similarity in the overall microbial communities among whales, these core members accounted for < 50% of the large intestine microbiota in about half of the whales, which suggests wide variation in the specific bacteria contributing to the functions of these core bacteria.

Our results revealed higher than expected abundances of wax esters in a portion of the samples collected from the colons. Together with the wide range in differences between forestomach and colon abundance in the paired samples (4–91%), it may be that wax ester digestion is more efficient in some whales than others. Moreover, the overall composition of the wax ester pool in the colon was significantly different from that of the stomach chambers, and yet all wax esters species in the colon were also observed in the stomachs, suggesting that the wax esters remaining in the colons represented undigested dietary wax esters. Wax ester digestibility, and the bioavailability of their hydrolytic products, may depend on a number of factors: the quantity of wax esters consumed, the quantity of triglycerides consumed with the wax esters, the concentration of bile salts (it has been proposed that triglycerides and bile salts both may improve the solubility of the highly hydrophobic wax esters), mechanisms for mixing, retention time, and/or chemical structure of the wax esters (chain length or degree of saturation) [52, 53, 55]. Given the correlation between wax esters and the microbiotas we observed in the jejunum (mid-small intestine) of bowhead whales, the presence or absence of certain microorganisms in the small intestine might also affect wax ester digestion and thus, the abundance of wax esters in the colon. It is possible that the higher than expected abundances of wax esters in some of the colon samples may be a result of any number of these factors; further studies are needed to identify the mechanism(s) for wax ester digestion in whales. Other lipids observed in large intestines generally comprised sterols and stanols and their esters, the pigment astaxanthin, quinones, small amounts of intact polar lipids, and diglycerides (see Supplementary Discussion).

Many of the lipids and microbes in the large intestine are excreted into the water column. Previous studies indicated that because marine mammals defecate in the photic zone, they contribute significantly to oceanic nutrient cycling and the stimulation of primary production, and hence, the oceans’ role in regulating atmospheric CO_2_ [1, 2]. Based on metagenomes of Australian sea lions (*Neophoca cinerea*) feces, Lavery et al. [61] proposed that marine mammal fecal microorganisms expedite the release of nutrients from fecal matter into the ocean, thereby increasing availability of certain nutrients for stimulating primary production. The function of the bowhead whale microbiotas was not examined in this study, but it is likely that bowhead whale fecal microbiotas may also function to release nutrients important to the marine food web from fecal particles. Moreover, the lipids excreted in the feces add important organic matter to the water column. Indeed, because wax esters play a major role in storing an abundance of the carbon fixed in the world’s oceans [10], the evidence suggesting digestion of wax esters revealed in our study indicates that whales and their microbes are likely contributing to carbon cycling in the oceans. Several whale populations in Alaska, including bowhead whales of the Bering–Chukchi–Beaufort Seas population, have recovered to near pre-exploitation numbers after the end of Yankee commercial whaling in the early 1900s and continue to grow [62, 63]. This increasing number of whales and their microbiotas would lead to digestion of more prey and release of more nutrients and carbon into the upper water column, thereby increasing biogeochemical cycling of carbon and nutrients in the high latitude seas.

### The coordination between the microbiotas and lipid digestion

Dietary lipids can profoundly influence the community composition and diversity of the intestinal microbiota [64, 65] and yet, conversely, the intestinal microbiota plays an essential role in the digestion and absorption of lipids [66], including microbiotas of the small intestine [67], the location where we observed significant decreases in prey lipids in bowhead whales. Intestinal bacteria also are intimately involved in host bile acid homeostasis [68] (bile acids are important for digestion of lipids, including wax esters). In our study, we observed a strong correlation between lipidome and microbiota with the progression of the GI tract. Based on the evidence in other systems, this correlation suggests that lipids in the bowhead diet may influence the community structure of the microbiota, and in turn, the microbiota may be involved in the digestion and absorption of the lipids. It is also possible that changes in the physicochemical properties across the GI tract contribute to the coordinated transformation of the lipids and microbiotas. While we cannot yet define the intricacies of the coordination, the lipidome–microbiota correlation likely represents a complex relationship driven by diet, microbiota composition and function, and host physiology.

### Considerations

A number of factors could have affected the composition of the microbiotas and lipidomes described in our study. First, the gut microbiota changes across the lifespan of mammals [69, 70]. Although this may also be the case for bowhead whales who have a lifespan estimated to be greater than 200 years [71], body length data indicates all but five of the sampled whales were sexually and physically immature juveniles that were likely only a few years old [72]. Three whales were likely young adults, and two were the aforementioned calves whose diets, lipidomes, and microbiotas were clearly distinct. Second, we observed a significant difference in microbial communities between years for a given anatomical site. These differences across years were likely a reflection of environmental factors, such as changes in diet; sample handling and analytical procedures were standardized and rigorously maintained to avoid year-to-year artifacts. Yet, difference in sample storage time may also have been a factor. Third, the time between death and sample collection was ~8–15 h. No signs of carcass decomposition were observed in our whales. As previously stated, 8–15 h is significantly less time than samples collected during necropsies of most large whales. While seawater and air temperatures during the towing of the whale to the butchering site and sample collection were near freezing and may have helped preserve the quality of the samples, bowhead whales are well insulated by a thick layer of blubber and thus, it is possible that the gut microbiotas and lipidomes may have been affected. Overall, if these factors were affecting the microbiotas and lipidomes, they would likely increase the variability in the data, which would make it more difficult to observe statistical differences.

## Conclusions

Diet has a substantial impact on the mammalian GI tract microbiota, which, in turn, has profound impacts on host health, including immune function, behavior, nutrition, and body fat condition [73–75]. We showed that microbes were associated with lipids in whales and propose that the whale microbiota may play a role in the digestion of lipids, particularly wax esters, which is not only crucial for the optimal nutrition and health needed for survival of individual whales and their species, but also may be integral to the cycling of these important molecules in the ocean. Sea ice melt and changes to climate are affecting the distribution, abundance, and nutritive quality of whales’ prey [76–80], which may, in turn, affect whale GI tract microbiotas. Changes to prey, along with climate driven changes to the physicochemical properties of the marine environment, almost certainly will affect suitability and quality of the habitats whales have used historically. Indeed, a shift in habitat use has been documented for a number of whale species, including bowhead whales [81] and the closely related endangered North Atlantic right whales (*Eubalaena glacialis*) [82]. With declines in health of North Atlantic right whales over the past 3 decades [83], it is important to understand whether or not the gut microbiotas of whales will have the metabolic flexibility to support their host’s health during such changes. Our results provide an important baseline from which to monitor potential changes to the bowhead whale gut microbiota and responses to any changes to quality and/or quantity of dietary lipids, thereby allowing a better understanding of if, and how, such alterations may impact the digestive processes of whales and their GI microbes, as well as nutrient cycling and stimulation of primary production in our oceans.

## Supplementary information

Supplementary Information
