# Supplementary material for: Coordinated transformation of the gut microbiome and lipidome of bowhead whales provides novel insights into digestion
Source: ISME J. 2019 Dec 2;14(3):688–701. doi: 10.1038/s41396-019-0549-y (PMC7031289; doi:10.1038/s41396-019-0549-y)
Supplement: Supplementary file 1 — Supplementary Information [file 41396_2019_549_MOESM1_ESM.docx]

ISME-J

Supplementary Information for

**Co-transformation of the gut microbiome and lipidome of bowhead whales provides novel insights into digestion**

Carolyn A. Miller, Henry Holm, Lara Horstmann, John C. George, Helen Fredricks, Benjamin A. S. Van Mooy, Amy Apprill

Carolyn A. Miller and Amy Apprill

Email: [cmiller@whoi.edu](mailto:cmiller@whoi.edu) and [aapprill@whoi.edu](mailto:aapprill@whoi.edu)

**This file includes:**

Figures S1 to S5

Tables S1 to S7

Supplementary Materials and Methods: provides further details of sequence, lipidomic, and data analyses.

Supplementary Discussion: provides further details of microbial sequence similarity with other marine mammals and lipids of the large intestine.

**Supplementary Figures and Tables**

Figure *S*1.

Figure *S*1. Boxplots of relative abundances of the phyla to which the 361 operational taxonomic units determined by minimum entropy decomposition [1] (MED nodes) belonged for each gastrointestinal tract location of bowhead whales. Five summary statistics are visualized in the boxplot: median, the two hinges, which correspond to the 25^th^ and 75^th^ percentiles (the first and third quartiles), and the upper and lower whiskers, which extend from the upper and lower hinges to the largest and smallest values no further than 1.5 times the inter-quartile range (the range between the hinges).

Figure *S*2.

Figure *S*2. Boxplots of abundances of each lipid class relative to total lipids for each gastrointestinal tract location of bowhead whales. Five summary statistics are visualized in the boxplot: median, the two hinges, which correspond to the 25^th^ and 75^th^ percentiles (the first and third quartiles), and the upper and lower whiskers, which extend from the upper and lower hinges to the largest and smallest values no further than 1.5 times the inter-quartile range (the range between the hinges).

Figure *S*3.

Figure *S*3. (A) Abundance of the wax esters class relative to total lipids in whales from which digesta was sampled from both forestomach and colon (*n* = 14) of bowhead whales. Diagonal lines between the forestomach and colon denote the paired data. Wax ester abundance in the forestomach is significantly greater than that in the colon (*P* < 0.0001). (B) Difference in wax ester abundance (relative to total lipids) between digesta samples from the forestomach and the colon in bowhead whales that were sampled in both locations. Five summary statistics are visualized in the boxplot: median, the two hinges, which correspond to the 25^th^ and 75^th^ percentiles (the first and third quartiles), and the upper and lower whiskers, which extend from the upper and lower hinges to the largest and smallest values no further than 1.5 times the inter-quartile range (the range between the hinges).

Figure *S*4.

Figure *S*4. Abundances of the triglyceride lipid class relative to total lipids in each gastrointestinal tract location of bowhead whales. Five summary statistics are visualized in the boxplot: median, the two hinges, which correspond to the 25^th^ and 75^th^ percentiles (the first and third quartiles), and the upper and lower whiskers, which extend from the upper and lower hinges to the largest and smallest values no further than 1.5 times the inter-quartile range (the range between the hinges). Asterisks indicate the forestomach through the duodenal ampulla are significantly different from both large intestine sites (*P* ≤ 0.05).

Figure *S5*.

Figure *S*5. Comparison of the bowhead whale microbiome across the gastrointestinal (GI) tract of bowhead whales with nursing calves included. Nonmetric multidimensional scaling (nMDS) comparisons of Bray-Curtis dissimilarities of microbial communities based on the 361 minimum entropy decomposition nodes [1] (MED nodes) (*n* = 123). Dispersion ellipse centroids are defined by the mean dissimilarities for each anatomical location and the ellipse shapes are defined by the covariances. Samples from nursing calves are labeled with their respective field identification numbers.

Figure *S*6.

Figure *S6*. Spearman correlations between log10 transformed relative abundance of select microbial taxa and relative abundance of the wax ester lipid class (relative to total lipids) in the jejunum, the mid-small intestine, of bowhead whales. Microbial taxa (operational taxonomic units generated by minimum entropy decomposition [1]; MED nodes) were selected by similarity percentages analysis (SIMPER) of Bray-Curtis dissimilarity as MEDs that significantly contributed to the dissimilarity among whales that had large differences between wax ester relative abundance in the forestomach and colon and those that had a small difference.

Table *S*1. Summary of bowhead whales sampled in Utqiaġvik, AK as part of the fall Alaska Native subsistence harvest, including gastrointestinal tract locations, sex, length, and year of collection.

| Whale_ID | Anatomical_Location | Sex | Length (cm) | Year |
| --- | --- | --- | --- | --- |
| W09B6 | Forestomach | Male | 990 | 2009 |
|  | Fundic chamber |  |  |  |
|  | Pyloric chamber |  |  |  |
|  | Duodenum |  |  |  |
|  | Colon |  |  |  |
| W09B7 | Forestomach | Female | 1130 | 2009 |
|  | Fundic chamber |  |  |  |
|  | Duodenum |  |  |  |
|  | Colon |  |  |  |
| W09B10 | Colon | Female | 890 | 2009 |
| W09B12 | Pyloric chamber | Female | 870 | 2009 |
|  | Duodenum |  |  |  |
|  | Colon |  |  |  |
| W11B8 | Forestomach | Female | 840 | 2011 |
|  | Duodenum |  |  |  |
|  | Jejunum |  |  |  |
|  | Ileum |  |  |  |
|  | Colon |  |  |  |
| W11B9 | Forestomach | Female | 1250 | 2011 |
|  | Fundic chamber |  |  |  |
|  | Pyloric chamber |  |  |  |
|  | Duodenum |  |  |  |
|  | Jejunum |  |  |  |
|  | Ileum |  |  |  |
|  | Ileocecal_Junction |  |  |  |
|  | Upper colon |  |  |  |
|  | Colon |  |  |  |
| W11B11 | Duodenum | Male | 850 | 2011 |
| W11B12 | Duodenum | Male | 1020 | 2011 |
|  | Colon |  |  |  |
| W11B13 | Forestomach | Male | 820 | 2011 |
|  | Colon |  |  |  |
| W11B14 | Duodenum | Male | 1170 | 2011 |
|  | Colon |  |  |  |
| W11B17 | Colon | Female | 1450 | 2011 |
| W11B18 | Colon | Female | 1020 | 2011 |
| W12B1 | Colon | Female | 1011 | 2012 |
| W12B2 | Colon | Female | 1008 | 2012 |
| W12B3 | Colon | Male | 988 | 2012 |
| W12B5 | Colon | Female | 790 | 2012 |
| W12B8 | Colon | Male | 831 | 2012 |
| W12B15 | Forestomach | Male | 838 | 2012 |
|  | Fundic chamber |  |  |  |
|  | Duodenal ampulla |  |  |  |
|  | Jejunum |  |  |  |
|  | Colon |  |  |  |
| W12B16 | Pyloric chamber | Male | 1031 | 2012 |
|  | Duodenum |  |  |  |
|  | Jejunum |  |  |  |
|  | Ileum |  |  |  |
|  | Upper colon |  |  |  |
|  | Colon |  |  |  |
| W12B17 | Colon | Female | 1082 | 2012 |
| W12B18 | Forestomach | Female | 940 | 2012 |
|  | Fundic chamber |  |  |  |
|  | Pyloric chamber |  |  |  |
|  | Jejunum |  |  |  |
|  | Ileum |  |  |  |
|  | Upper colon |  |  |  |
|  | Colon |  |  |  |
| W12B19 | Forestomach | Male | 942 | 2012 |
|  | Fundic chamber |  |  |  |
|  | Pyloric chamber |  |  |  |
|  | Duodenal ampulla |  |  |  |
|  | Duodenum |  |  |  |
|  | Jejunum |  |  |  |
|  | Ileum |  |  |  |
|  | Colon |  |  |  |
| W12B20 | Duodenum | Male | 889 | 2012 |
|  | Ileum |  |  |  |
|  | Upper colon |  |  |  |
|  | Colon |  |  |  |
| W12B21 | Forestomach | Female | 1334 | 2012 |
|  | Duodenum |  |  |  |
|  | Colon |  |  |  |
| W12B22 | Colon | Female | 919 | 2012 |
| W12B23 | Forestomach | Female | 848 | 2012 |
| W12B24 | Colon | Male | 1013 | 2012 |
| W13B3 | Forestomach | Male | 790 | 2013 |
|  | Fundic chamber |  |  |  |
|  | Pyloric chamber |  |  |  |
|  | Duodenal ampulla |  |  |  |
|  | Duodenum |  |  |  |
|  | Jejunum |  |  |  |
|  | Ileum |  |  |  |
|  | Upper colon |  |  |  |
|  | Colon |  |  |  |
| W13B4 | Duodenum | Male | 1171 | 2013 |
|  | Upper colon |  |  |  |
|  | Colon |  |  |  |
| W13B6 | Forestomach | Female | 937 | 2013 |
|  | Fundic chamber |  |  |  |
|  | Duodenal ampulla |  |  |  |
|  | Duodenum |  |  |  |
|  | Jejunum |  |  |  |
|  | Ileum |  |  |  |
|  | Upper colon |  |  |  |
|  | Colon |  |  |  |
| W13B7 | Forestomach | Female | 859 | 2013 |
|  | Fundic chamber |  |  |  |
|  | Pyloric chamber |  |  |  |
|  | Duodenal ampulla |  |  |  |
|  | Duodenum |  |  |  |
|  | Ileum |  |  |  |
|  | Upper colon |  |  |  |
|  | Colon |  |  |  |
| W13B8 | Fundic chamber | Female | 678 | 2013 |
|  | Jejunum |  |  |  |
|  | Colon |  |  |  |
| W13B9 | Forestomach | Female | 922 | 2013 |
|  | Jejunum |  |  |  |
|  | Upper colon |  |  |  |
|  | Colon |  |  |  |
| W13B10 | Forestomach | Male | 1138 | 2013 |
|  | Jejunum |  |  |  |
|  | Upper colon |  |  |  |
|  | Colon |  |  |  |
| W13B11 | Forestomach | Male | 1133 | 2013 |
|  | Colon |  |  |  |
| W13B12 | Forestomach | Female | 864 | 2013 |
|  | Jejunum |  |  |  |
|  | Colon |  |  |  |
| W13B13 | Stomach | Male | 610 | 2013 |
|  | Jejunum |  |  |  |
|  | Colon |  |  |  |
| W13B14 | Forestomach | Male | 919 | 2013 |
|  | Jejunum |  |  |  |
|  | Colon |  |  |  |

Table *S*2. Gradient profiles for liquid chromatography for lipidomics analysis of digesta samples from bowhead whale gastrointestinal tracts.

| Time, minutes | Flow rate, mL min^-1^ | A, % |
| --- | --- | --- |
| 0.0 | 0.4 | 5 |
| 30.0 | 0.4 | 50 |
| 30.1 | 0.4 | 5 |
| 40 | 0.4 | 5 |

Table *S*3. Eluents for liquid chromatography for lipidomics analysis of digesta samples from bowhead whale gastrointestinal tracts.

| *Solvent / reagent* | *A* | *B* |
| --- | --- | --- |
| Hexane, > 65% n-hexane, GC Resolv grade. # H307, Thermo Fisher Scientific. | 80 | 0 |
| Iso-propanol, # A461, Optima LCMS grade, Thermo Fisher Scientific. | 20 | 90 |
| Water, fresh Milli-Q, EMD Millipore. Billerica, MA, USA | 0 | 10 |
| Formic acid, LCMS grade, # 11202, Covachem, Loves Park, IL, USA. | 0.1 | 0.1 |
| Ammonium hydroxide, ≥ 25% NH_3_(aq), LCMS grade. # 44273, Fluka, Thermo Fisher Scientific. | 0.04 | 0.04 |

Table *S*4. External standards used for ionization response corrections for the major lipid classes.

| Peak groups | Standard [Source] |
| --- | --- |
| Wax esters | *n*-Hexadecyl-1,1,2,2-d_4_ Hexadecanoate-16,16,16-d_3_ [CDN Isotopes, Inc., Pointe-Claire, QC, Canada] |
| Sterol and stanol esters | 5-Cholestene 3-palmitate [Sigma Aldrich, Burlington, MA, USA] |
| Quinone | ubiquinone-10 [Sigma Aldrich, Burlington, MA, USA] |
| Astaxanthin | Astaxanthin [Sigma Aldrich, Burlington, MA, USA] |
| Diacylglyceryl hydroxymethyl-trimethyl-β-alanine | deuterated 1,2-dipalmitoyl-sn-glycero-3-O-4'-[N,N,N-trimethyl(d9)]-homoserine (DGTS-d9) [Avanti Polar Lipids, Inc., Alabaster, AL, USA] |
| Diacylglyceryl-carboxy-N-hydroxymethyl-choline | deuterated 1,2-dipalmitoyl-sn-glycero-3-O-4'-[N,N,N-trimethyl(d9)]-homoserine (DGTS-d9) [Avanti Polar Lipids, Inc., Alabaster, AL, USA] |
| Phosphatidylcholine | deuterated 1,2-dipalmitoyl-sn-glycero-3-O-4'-[N,N,N-trimethyl(d9)]-homoserine (DGTS-d9) [Avanti Polar Lipids, Inc., Alabaster, AL, USA] |
| Phosphatidylethanolamine | 1-pentadecanoyl-2-oleoyl(d_7_)-sn-glycero-3-phosphoethanolamine (15:0-18:1-d_7_ PE) [Avanti Polar Lipids, Inc., Alabaster, AL, USA] |
| Glycolipids | D-glucosyl-ß-1,1'-N-stearoyl-D-erythro-sphingosine-d_5_ (C18 Glucosyl(ß) Ceramide-d_5_) [Avanti Polar Lipids, Inc., Alabaster, AL, USA] |
| Diglyceride | 1-pentadecanoyl-2-oleyol(d_7_)-sn-glycerol [Avanti Polar Lipids, Inc., Alabaster, AL, USA] |
|  |  |

Table *S*5. Pairwise comparisons of bowhead whale gastrointestinal tract microbial community composition between gastrointestinal tract locations and sampling year according to PERMANOVA analysis of Bray-Curtis dissimilarity.

| Comparison |  | df | SS | Average similarity between GI locations (%) | Pseudo-F^f^ or t - value | p-value |
| --- | --- | --- | --- | --- | --- | --- |
|  |  |  |  |  |  |  |
| Anatomical Locations | | 8 | 1.69E+05 |  | 12.803^f^ | 0.001* |
| Pariwise comparisons: | |  |  |  |  |  |
| Forestomach | Fundic chamber |  |  | 47 | 0.99437 | 0.439 |
| Forestomach | Pyloric chamber |  |  | 44 | 1.1552 | 0.246 |
| Forestomach | Duodenal ampulla |  |  | 41 | 1.2833 | 0.129 |
| Forestomach | Duodenum |  |  | 30 | 2.429 | 0.001* |
| Forestomach | Jejunum |  |  | 22 | 3.0255 | 0.001* |
| Forestomach | Ileum |  |  | 16 | 4.0661 | 0.001* |
| Forestomach | Upper colon |  |  | 8 | 5.8333 | 0.001* |
| Forestomach | Colon |  |  | 7 | 7.4727 | 0.001* |
| Fundic chamber | Pyloric chamber |  |  | 47 | 0.74009 | 0.797 |
| Fundic chamber | Duodenal ampulla |  |  | 44 | 0.92328 | 0.514 |
| Fundic chamber | Duodenum |  |  | 32 | 1.9112 | 0.004* |
| Fundic chamber | Jejunum |  |  | 24 | 2.4302 | 0.001* |
| Fundic chamber | Ileum |  |  | 18 | 3.5949 | 0.001* |
| Fundic chamber | Upper colon |  |  | 9 | 5.4933 | 0.001* |
| Fundic chamber | Colon |  |  | 8 | 6.1071 | 0.001* |
| Pyloric chamber | Duodenal ampulla |  |  | 44 | 0.68703 | 0.779 |
| Pyloric chamber | Duodenum |  |  | 32 | 1.4909 | 0.054 |
| Pyloric chamber | Jejunum |  |  | 24 | 2.0612 | 0.001* |
| Pyloric chamber | Ileum |  |  | 17 | 3.221 | 0.001* |
| Pyloric chamber | Upper colon |  |  | 7 | 4.9922 | 0.001* |
| Pyloric chamber | Colon |  |  | 6 | 5.5285 | 0.001* |
| Duodenal ampulla | Duodenum |  |  | 31 | 1.2422 | 0.164 |
| Duodenal ampulla | Jejunum |  |  | 24 | 1.5647 | 0.021* |
| Duodenal ampulla | Ileum |  |  | 18 | 2.6033 | 0.003* |
| Duodenal ampulla | Upper colon |  |  | 7 | 4.2681 | 0.001* |
| Duodenal ampulla | Colon |  |  | 7 | 4.4205 | 0.001* |
| Duodenum | Jejunum |  |  | 29 | 1.1673 | 0.2 |
| Duodenum | Ileum |  |  | 25 | 2.3167 | 0.001* |
| Duodenum | Upper colon |  |  | 15 | 3.8237 | 0.001* |
| Duodenum | Colon |  |  | 14 | 5.2292 | 0.001* |
| Jejunum | Ileum |  |  | 31 | 1.61 | 0.015* |
| Jejunum | Upper colon |  |  | 22 | 3.1654 | 0.001* |
| Jejunum | Colon |  |  | 21 | 4.2229 | 0.001* |
| Ileum | Upper colon |  |  | 43 | 2.4445 | 0.004* |
| Ileum | Colon |  |  | 39 | 2.7549 | 0.002* |
| Upper colon | Colon |  |  | 58 | 0.96077 | 0.464 |
|  |  |  |  |  |  |  |
| Year nested within anatomical location | | 22 | 44680 |  | 1.3477^f^ | 0.019* |
| Pairwise comparisons for colon samples (n) | | |  |  |  |  |
| 2009 (4) | 2011 (7) |  |  | 53 | 1.8391 | 0.022* |
| 2009 | 2012 (14) |  |  | 48 | 1.3917 | 0.088 |
| 2009 | 2013 (11) |  |  | 50 | 1.9759 | 0.007* |
| 2011 | 2012 |  |  | 53 | 1.112 | 0.279 |
| 2011 | 2013 |  |  | 55 | 1.7564 | 0.015* |
| 2012 | 2013 |  |  | 51 | 1.3793 | 0.08 |
|  |  |  |  |  |  |  |
| Sex nested within anatomical location | | 9 | 17315 |  | 1.2783^f^ | 0.109 |

* p < 0.05

Table *S*6. Pairwise comparisons of bowhead whale gastrointestinal tract lipidome between gastrointestinal tract locations according to PERMANOVA analysis of Bray-Curtis dissimilarity.

| Comparison |  | df | SS | Average similarity between GI locations (%) | Pseudo-F^f^ or t - value | p-value |
| --- | --- | --- | --- | --- | --- | --- |
|  |  |  |  |  |  |  |
| Anatomical Locations | | 7 | 4.90E+04 |  | 10.452 | 0.001* |
| Pairwise comparisons: | |  |  |  |  |  |
| Forestomach | Fundic chamber |  |  | 62 | 0.77547 | 0.661 |
| Forestomach | Duodenal ampulla |  |  | 63 | 1.3654 | 0.103 |
| Forestomach | Duodenum |  |  | 52 | 2.3893 | 0.001* |
| Forestomach | Jejunum |  |  | 48 | 3.6327 | 0.001* |
| Forestomach | Ileum |  |  | 46 | 3.8788 | 0.001* |
| Forestomach | Upper colon |  |  | 44 | 4.9023 | 0.001* |
| Forestomach | Colon |  |  | 41 | 6.4768 | 0.001* |
| Fundic chamber | Duodenal ampulla |  |  | 64 | 0.80809 | 0.579 |
| Fundic chamber | Duodenum |  |  | 54 | 1.4396 | 0.101 |
| Fundic chamber | Jejunum |  |  | 52 | 2.4145 | 0.002* |
| Fundic chamber | Ileum |  |  | 49 | 2.8403 | 0.001* |
| Fundic chamber | Upper colon |  |  | 46 | 3.8703 | 0.001* |
| Fundic chamber | Colon |  |  | 43 | 4.6754 | 0.001* |
| Duodenal ampulla | Duodenum |  |  | 60 | 0.90808 | 0.517 |
| Duodenal ampulla | Jejunum |  |  | 57 | 1.9891 | 0.009* |
| Duodenal ampulla | Ileum |  |  | 54 | 2.7613 | 0.002* |
| Duodenal ampulla | Upper colon |  |  | 50 | 3.9439 | 0.001* |
| Duodenal ampulla | Colon |  |  | 47 | 3.9544 | 0.001* |
| Duodenum | Jejunum |  |  | 57 | 1.3587 | 0.096 |
| Duodenum | Ileum |  |  | 55 | 1.8873 | 0.009* |
| Duodenum | Upper colon |  |  | 51 | 2.9302 | 0.001* |
| Duodenum | Colon |  |  | 48 | 4.2541 | 0.001* |
| Jejunum | Ileum |  |  | 66 | 1.216 | 0.192 |
| Jejunum | Upper colon |  |  | 61 | 2.7224 | 0.001* |
| Jejunum | Colon |  |  | 56 | 3.635 | 0.001* |
| Ileum | Upper colon |  |  | 69 | 1.974 | 0.013* |
| Ileum | Colon |  |  | 63 | 2.5778 | 0.001* |
| Upper colon | Colon |  |  | 72 | 1.1095 | 0.281 |

*p < 0.05

Table *S*7. Average Bray-Curtis similarity within gastrointestinal tract sampling location for the microbial communities and the lipidomes of bowhead whales.

|  |  |  | Average similarity within GI location (%) | |
| --- | --- | --- | --- | --- |
| Anatomical Area | Anatomical Locations |  | Microbiotas (n) | Lipidome (n) |
| Stomachs | Forestomach |  | 46.2 (18) | 63.4 (16) |
|  | Fundic chamber |  | 48.4 (10) | 58.8 (8) |
|  | Pyloric chamber |  | 42.6 (8) |  |
| Small Intestine | Duodenal ampulla |  | 37.7 (5) | 67.0 (5) |
|  | Duodenum |  | 30.7 (16) | 54.4 (15) |
|  | Jejunum |  | 28.1 (14) | 64.5 (11) |
|  | Ileum |  | 45.9 (9) | 70.2 (8) |
| Large Intestine | Upper colon |  | 63.8 (10) | 77.2 (10) |
|  | Colon |  | 52.7 (36) | 69.5 (31) |

**Supplementary Materials and Methods**

*Sequence analysis*

Mothur v.1.33.3 [ref.-2] was used to assemble, denoise, and quality filter the raw reads. Primer and barcodes were removed and reads joined to generate a total of 10 813 369 sequences with an average length of 258 base pairs. After sequences were trimmed and ambiguous base pair calls were removed, a total of 8 675 119 reads of an average of 252 base pairs were classified using a k-nearest neighbor consensus algorithm in Mothur with the Silva ribosomal RNA sequence database (v.119) [3]. Sequences identified as chloroplasts (1196), mitochondria (14), and Eukaryota (27) were removed from the dataset. Within Mothur, chimeras were detected using UCHIME [4] and 36,236 chimeras (0.42% of the dataset) were removed. The sequence dataset was subsampled to 12 000 reads per sample to minimize any effect from read count variation and control samples were removed.

*Mass spectrometry analysis*

For all analytical runs, the mass spectrometer was programmed for a full scan (350 - 2000 m/z positive, 200 – 2000 m/z negative) followed by data-dependent tandem MS^2^ (top 3 most abundant ions) alternating between positive and negative ion modes, with an isolation window for MS^2^ scans of 2.0 m/z. Stepped normalized collision energy of 25, 50, and 75 ensured adequate fragmentation for all lipid species. Dynamic exclusion mode was used with a 10 second time window meaning that any ion previously analyzed by MS^2^ would not be analyzed again within that time. This allowed for a breadth of data while still obtaining MS^2^ scans at the chromatographic peak apex. The mass spectrometer had a heated electrospray source set to 100 °C, and the capillary temperature set to 200 °C. Spray voltages were 4.5 kV (+) and 3.5 kV (-), and both the sheath gas and auxiliary gas flows were set to 20 (arbitrary units). The resolution was set to the maximum of 140 000 (at 200 m/z) for full scan, giving a resolution for a wax ester of m/z 634.6497 of ~90 000. The resolution for MS^2^ scans was set to 17,500 shortening the time for each MS^2^ scan and thus for 20-30 points across a peak. To maintain good mass accuracy, so-called ‘lock masses’ were used where non-analyte ions are used to continuously correct the mass calibration during each run. The lock masses used are listed below in Supplementary Methods Table *S*8. Siloxanes are frequently observed as background contamination in LCMS analyses and being well characterized, they make good lock masses. However, we found that siloxane concentrations varied depending on the sample history and cleanliness of the system. To ensure a reliable source and suitable concentration of the siloxanes a PTFE/red silicone rubber septum (Agilent # 5182-0731) was placed in the source housing of the Q Exactive before starting each batch of analyses. No suitable background contaminant ion for lock mass in negative ion mode was identified, so a small glass vial containing ~100mg of nonadecanoic acid was also placed inside the source housing. The volatility of the n-C19 fatty acid is such that a low but steady signal of ion 297 m/z is observed in negative ion mode. Mass accuracy was typically around 1 ppm, for example triacylglyceride C48:1 (822.7545, C_51_H_100_O_6_N^+^) was observed to have a mass accuracy of 0.57 ppm.

Supplementary Methods Table *S*8. Lock masses for the mass spectrometer for lipidomics analysis of digesta samples from bowhead whale gastrointestinal tracts.

| m/z | Chemical formula | Polarity | Compound |
| --- | --- | --- | --- |
| 536.16591 | C_14_H_46_NO_7_Si_7_^+^ | Positive | cyclodimethylpolysiloxane Si = 7 (ammonium adduct) |
| 610.18416 | C_16_H_52_NO_8_Si_8_^+^ | Positive | cyclodimethylpolysiloxane Si = 8 (ammonium adduct) |
| 684.20350 | C_18_H_58_NO_9_Si_9_^+^ | Positive | cyclodimethylpolysiloxane Si = 9 (ammonium adduct) |
| 297.27990 | C_19_H_37_O_2_^-^ | Negative | n-C19 fatty acid (M-H) |

*Data analysis*

Non-metric multidimensional scaling analyses were conducted using metaMDS in the *vegan* package (v 2.5-2) [ref.-5] in RStudio [6] for R [7] with the Bray-Curtis index as described by Eren et al. [1]. Covariances were calculated using the cov.wt function in the stats package in R and the shapes were overlaid on the figure by calling the veganCovEllipse function of the *vegan* package. The *vegan* package was also used to conduct the Mantel tests. The *stats* package (v3.4.0) in R was used to conduct the Spearman’s correlation, Kruskal-Wallis rank sum tests, and the Pairwise t-tests. Dunn’s test was conducted in R using *dunn.test* package (v1.3.5). The ggplot2 package in R (v3.1.0) [ref.-8] was used for all figures. The *Phyloseq* package in R (v1.20.0) [ref.-9] was used for alpha diversity analysis of the microbiome and, along with *dplyr* (v0.7.6) [ref.-10], as the data organizational framework from which to conduct the analyses. Permutational analyses of variance (PERMANOVA) were conducted using the PERMANOVA add-on to the Primer 7 software (PRIMER-E, Ltd, Plymouth, UK) to test for differences in microbial community composition and lipidome among GI tract locations, and, for the microbiome among sampling years, which was tested as nested within anatomical location. PERMANOVA tests were conducted using Bray-Curtis similarities of square root transformed relative abundances with 999 unrestricted permutations under Type III partial sums of squares model. Monte Carlo simulations were used to confirm significant differences. Similarity percentages analyses were also conducted using Primer 7.

**Supplementary Discussion**

*Microbes of the stomach chambers share similarity with other cetaceans*

The bacteria we identified in the stomachs of bowhead whales were similar to those previously isolated from the GI tracts minke whales (*Balaenoptera acutorostrata*) [*Fusobacterium*, *Peptostreptococcus*, *Cetobacterium*, Lactobacillales; 11, 12], harbor porpoise (*Phocoena phocoena*) [*Cetobacterium*; 12] and to sequences recovered from bottlenose dolphins (*Tursiops truncatus*) [*Fusobacterium* and *Actinobacillus*; 13]. Moreover, the exoskeletons of bowhead whale prey are rich in the polysaccharide chitin and some bacterial strains in the genus *Lactococcus*, the genus to which one of our core bacteria belonged, exhibit the ability to ferment chitin [14].

*Core bacterial taxa of the small intestine*

Four of the five other bacterial groups that emerged as core members (>1% abundance in >50% of samples) of the bowhead whale small intestine microbiotas were anaerobic members of the Clostridia class of Firmicutes (three *Clostridium*, and one *Romboutsia*). The fifth group was a member of Fusobacteria (*Fusobacterium*). *Clostridium* (MED2594), which emerged as a core member in the ileum and remained a core member of the large intestine microbiota, was 100% similar in partial SSU rRNA gene sequence composition to *Clostridium paraputrificum*, a known chitin fermenter [15]. *Romboutsia* are well-established intestinal bacteria, with diverse metabolic capabilities that include fermentation of single amino acids [16, 17]. The other *Clostridium* and *Fusobaterium* identified here share strong sequence similarity to bacteria in mammalian feces, but no specific function is known that would elucidate their role in the bowhead whale small intestine.

*Core bacterial taxa of the colon share similarity with sequences recovered from pinnipeds*

Bacterial taxa that emerged as core members (>1% abundance in >50% of samples) in the microbiotas of bowhead whale colons were 99% similar to sequences previously found in the colons of seals and sea lions as follows: (a) California sea lion (*Zalophus californianus*) [JQ207789; ref-13] (MED3810 of family Erysipelotrichaceae); (b) Antarctic fur seals (*Arctocephalus gazelle*) [MH728290; ref-18], gray seals (*Halichoerus grypus*) [GQ867460; ref-19], Weddell seals (*Leptonychotes weddellii*) [KM100430; ref-20] and California sea lions [JQ208488; ref-13] (MED3596, *Alloprevotella*); (c) California sea lions [JQ207342; ref-13] and gray seals [GQ867499; ref-19] (MED 604, *Eubacterium*).

*Lipids of the Large Intestine*

Wax esters, sterols and stanols and their esters, astaxanthin (a pigment), quinones, small amounts of intact polar lipids, and diglycerides were observed in the large intestine. Interestingly, the large intestine contained slightly less sterols and slightly more stanols than the small intestine, which may be, in part, a result of microbial conversion of cholesterol into coprostanol, as noted in other vertebrates [21-23]. Indeed, coprostanol was documented in fecal samples of baleen whales and used as a marker to study marine mammal habitats [24].

**References for Supplementary Information**

1. Eren AM, Morrison HG, Lescault PJ, Reveillaud J, Vineis JH, Sogin ML. Minimum entropy decomposition: unsupervised oligotyping for sensitive partitioning of high-throughput marker gene sequences. ISME J. 2015; 9(4):968-79.

2. Kozich JJ, Westcott SL, Baxter NT, Highlander SK, Schloss PD. Development of a Dual-Index Sequencing Strategy and Curation Pipeline for Analyzing Amplicon Sequence Data on the MiSeq Illumina Sequencing Platform. Appl Environ Microbiol. 2013; 79(17):5112-20.

3. Pruesse E, Quast C, Knittel K, Fuchs BM, Ludwig W, Peplies J, et al. SILVA: a comprehensive online resource for quality checked and aligned ribosomal RNA sequence data compatible with ARB. Nucleic Acids Res. 2007; 35(21):7188-96.

4. Edgar RC, Haas BJ, Clemente JC, Quince C, Knight R. UCHIME improves sensitivity and speed of chimera detection. Bioinformatics. 2011; 27(16):2194-200.

5. Oksanen J, Blanchet FG, Kindt R, Legendre P, Minchin PR, O’Hara R, et al. vegan: Community Ecology Package. R package version 2.3-1. 2015.

6. RStudio Team. RStudio: Integrated Development for R. 1.0.143 ed. Boston, MA: RStudio, Inc.; 2016.

7. R Core Team. R: A language and environment for statistical computing. 3.4.0 ed. Vienna, Austria: R Foundation for Statistical Computing; 2017.

8. Wickham H. ggplot2: elegant graphics for data analysis. R package version 3.1.0 ed: Springer Science & Business Media; 2009.

9. McMurdie PJ, Holmes S. phyloseq: an R package for reproducible interactive analysis and graphics of microbiome census data. PloS one. 2013; 8(4):e61217.

10. Wickham H, Francois R, Henry L, Müller K. dplyr: A grammar of data manipulation. R package version 0.7.2. ed2017.

11. Olsen MA, Aagnes TH, Mathiesen SD. Digestion of herring by indigenous bacteria in the minke whale forestomach. Appl Environ Microbiol. 1994; 60(12):4445-55.

12. Foster G, Ross H, Naylor R, Collins M, Ramos CP, Garayzabal FF, et al. *Cetobacterium ceti* gen. nov., sp. nov., a new Gram‐negative obligate anaerobe from sea mammals. Lett Appl Microbiol. 1995; 21(3):202-6.

13. Bik EM, Costello EK, Switzer AD, Callahan BJ, Holmes SP, Wells RS, et al. Marine mammals harbor unique microbiotas shaped by and yet distinct from the sea. Nat Commun. 2016; 7:10516.

14. Vaaje‐Kolstad G, Bunæs AC, Mathiesen G, Eijsink VG. The chitinolytic system of *Lactococcus lactis* ssp. *lactis* comprises a nonprocessive chitinase and a chitin‐binding protein that promotes the degradation of α‐and β‐chitin. FEBS J. 2009; 276(8):2402-15.

15. Evvyernie D, Yamazaki S, Morimoto K, Karita S, Kimura T, Sakka K, et al. Identification and characterization of *Clostridium paraputrificum* M-21, a chitinolytic, mesophilic and hydrogen-producing bacterium. J Biosci Bioeng. 2000; 89(6):596-601.

16. Gerritsen J, Hornung B, Renckens B, van Hijum SA, dos Santos VAM, Rijkers GT, et al. Genomic and functional analysis of *Romboutsia ilealis* CRIBT reveals adaptation to the small intestine. PeerJ. 2017; 5:e3698.

17. Gerritsen J, Hornung B, Staneva I, Ritari J, Paulin L, Rijkers GT, et al. Comparative genomics and functional analysis of the genus *Romboutsia* provides insight into adaptation to an intestinal lifestyle. In: Gerritsen J, editor. The genus *Romboutsia*: genomic and functional characterization of novel bacteria dedicated to life in the intestinal tract. Wageningen: Doctoral Dissertation. Wageningen University; 2015. p. 187-230.

18. Grosser S, Sauer J, Paijmans AJ, Caspers BA, Forcada J, Wolf JB, et al. Fur seal microbiota are shaped by the social and physical environment, show mother‐offspring similarities and are associated with host genetic quality. Mol Ecol. 2019.

19. Glad T, Kristiansen VF, Nielsen KM, Brusetti L, Wright A-DG, Sundset MA. Ecological characterisation of the colonic microbiota in Arctic and sub-Arctic seals. Microb Ecol. 2010; 60(2):320-30.

20. Banks JC, Cary SC, Hogg ID. Isolated faecal bacterial communities found for Weddell seals, *Leptonychotes weddellii*, at White Island, McMurdo Sound, Antarctica. Polar Biol. 2014; 37(12):1857-64.

21. Eyssen HJ, Parmentier GG, Compernolle FC, de Pauw G, Piessens‐Denef M. Biohydrogenation of sterols by *Eubacterium* ATCC 21,408—*nova* species. Eur J Biochem. 1973; 36(2):411-21.

22. Leeming R, Ball A, Ashbolt N, Nichols P. Using faecal sterols from humans and animals to distinguish faecal pollution in receiving waters. Water Res. 1996; 30(12):2893-900.

23. Martin W, Ravi Subbiah M, Kottke B, Birk C, Naylor M. Nature of fecal sterols and intestinal bacterial flora. Lipids. 1973; 8(4):208-15.

24. Venkatesan M, Santiago C. Sterols in ocean sediments: novel tracers to examine habitats of cetaceans, pinnipeds, penguins and humans. Mar Biol. 1989; 102(4):431-7.
